# Historical perspective of code clone refactorings in evolving software

**DOI:** 10.1371/journal.pone.0277216

**Published:** 2022-12-01

**Authors:** Jaweria Kanwal, Onaiza Maqbool, Hamid Abdul Basit, Muddassar Azam Sindhu, Katsuro Inoue

**Affiliations:** 1 Software Engineering Dept., National University of Moderns Languages, Islamabad, Pakistan; 2 Computer Science Dept., Quaid-i-Azam University, Islamabad, Pakistan; 3 College of Computer and Information Sciences, Prince Sultan University, Riyadh, Saudi Arabia; 4 Graduate School of Information Science and Technology, Osaka University, Osaka, Japan; University of Pisa, ITALY

## Abstract

Cloning in software is generally perceived as a threat to its maintenance and that is why it needs to be managed properly. Understanding clones from a historical perspective is essential for effective clone management. Analysis of code refactorings performed on clones in previous releases will help developers in taking decisions about clone refactoring in future releases. In this paper we perform a longitudinal study on the evolution of clone refactorings in various versions of five software systems. To perform a systematic study on clone refactoring evolution, we define clone evolution patterns for studying refactorings in a formal notation. Our results show that only a small proportion of code clones are refactored between the versions and most of the refactorings are inconsistent within clone classes. Moreover, clone refactorings may cause clone removal. Analysis of the source code of refactored clones reveals similar reasons of inconsistent refactorings and clone removal for five Java systems. This analysis will help in devising appropriate strategies for managing clone refactorings in software and hence provide foundation for devising better clone management tools.

## Introduction

Code clones are the code fragments in a software that are identical or similar to each other. Clones arise in a software as a result of developers’ copy-paste activity during software development. There are various reasons of cloning such as code reuse, programming language limitations and implementation of similar features during development [1]. Code cloning is a common phenomenon and clones exist in almost all kinds of software systems. Empirical studies show that generally software contains 9%-17% of code clones [1].

In the literature there are two different views about the use of clones in software development. On the one hand, clones are considered harmful for the system and represent poor quality. Redundant code increases the size of the software which requires extra resources. Clones are also considered risky from the maintenance point of view e.g. making changes in some code fragments and leaving their counterparts will result in inconsistency within the system [2, 3]. On the other hand, cloning of existing code provides some benefits e.g. reuse of tested code which reduces development cost for reinventing the wheel [4] and facilitating independent evolution for similar feature implementation. In early research on code clones, the focus was on detection of code clones in a system and proposing methods for their removal [5]. With time it has been realized that the removal of clones may not be an appropriate solution since clones are not necessarily bad. Recent research on code clones show that ““cloning considered harmful” considered harmful” [6]. Thus focus has shifted to clone management. Clone management deals with the analysis of code clones so that positive effects of cloning can be exploited and risks can be minimized [7].

Refactoring of clones is an important maintenance activity to improve code quality. Refactoring is the process of improving the internal structure of a software system without affecting its functional behavior [8]. The aim of code refactoring is to enhance software maintainability and code quality by removing design flaws [9]. There are different types of refactoring that provide solutions for different types of programming problems. Most well known refactoring patterns have been proposed by Fowler [8]. There are many clone detection and analysis tools [10, 11] that help developers in refactoring clones but little work has been done on investigating code clones in terms of refactoring performed on them in previous releases. In literature it is shown that developers rely on history of clones to decide about their refactoring instead of their present situation [12].

Analysis of clone refactorings in previous releases offers strong insights which help developers in taking decisions in future software development. For example, if a maintainer is given a task of identifying possible clone refactorings in a system during its evolution, analysis of previously refactored clones will help in selecting clones for refactoring. Another scenario is when a developer is given a task of refactoring a clone class. A developer needs some information such as whether it is refactorable or not, which refactoring techniques can be applied on it, whether same refactoring technique can be applied on all clone instances. Answering these questions requires a lot of manual analysis because there is no tool support for automatic analysis of clone refactorings. Characteristics of clone classes that are refactored in previous releases will guide maintainers in current clone refactoring task.

In our previous work [13], we investigated how frequently code clones are refactored between releases. In this paper, we extend our study by performing a longitudinal study on five Java systems to investigate code clone refactoring performed on them between the releases. In this paper, our main goal is to understand evolutionary characteristics of clone refactorings by studying their prevalence, their impact and developer’s behavior while performing refactoring on clones during software evolution. We enhance the study by formally defining evolution patterns for clones refactoring so that evolution of clone refactorings can be analyzed more effectively. Further we present our approach in detail, include more versions of five subject systems and provide detailed discussion of the results. Our major contributions in this paper are as follows:

1) We present formal definition of clone evolution patterns in terms of refactoring. Clone evolution patterns have been defined in literature [2] for studying clone changes in versions, however, for studying evolution in clone refactorings, they need to be redefined as clone refactoring represent specific changes that make improvements in the code.
2) We present a detailed study of clone refactoring evolution by considering multiple versions of five well-known subject systems. We study the evolution of clone refactoring by answering the following research questions:
*RQ1: What ratio of code clones are refactored between the releases in software systems?*

Ratio of refactored clones will help developers in assessing how to handle clones during software evolution i.e. a high ratio may indicate that developers consider clones harmful for the software and perform rigorous refactorings on them. An analysis of refactored clones during releases will also help in identifying characteristics of software clones such as which type of clones are usually considered for refactoring by developers and also which refactoring types are applied on them.

*RQ2: Does code refactoring remove clones from the system?*

We analyze how clone refactorings impact the cloning in a software i.e. whether refactoring performed on code clones cause clone removal from the system or the clone refactorings are applied as regular maintenance activity for code improvements. Analysis of refactoring types applied on removed clones will indicate how code clones are removed from the system, i.e. either clones are removed with clone removal refactoring only or other refactoring types also cause clone removal. This analysis will help in understanding characteristics of removed clones which will help in suggesting clone candidates for removal in future.

*RQ3: Are clones refactored consistently/inconsistently during software evolution?*

An analysis of consistent clone refactoring will help in understanding how maintainers deal with cloning in the system. For example if all clones of a certain clone group are applied same refactoring tasks then it indicates that developers are well aware about clones and managing them properly. On other hand an inconsistent refactoring of clones may lead to some hidden bug propagation in the system. An analysis of clone refactoring performed in software code will help in understanding the reasons of consistent/inconsistent refactoring at implementation level.

3) We developed a tool *CRefEvol* (Evolution patterns of clone refactorings), “https://github.com/j-kanwal/CRefEvol”, to evaluate the usefulness of our proposed approach in a development environment. A variety of tools are available to analyze clones in versions but they don’t analyze evolution of clone refactorings. Our tool extracts and visualizes evolution patterns of clone refactorings in various versions of a software.

There are some related research studies [7, 14] on clone refactorings but the focus of these studies is on the investigation of clone removal i.e. how developers remove clones from the system through refactoring techniques. Our work is different in two aspects. First, we studied clone refactorings by taking a broader view e.g. we include all refactoring techniques in our analysis that are applied on clones in previous releases. This analysis will reveal how different refactoring techniques make changes in cloned code in a software. Secondly, we perform a longitudinal study on clone refactorings through different clone evolution patterns that reveal how clones evolve in their lifetime and how developers treat clones when they perform refactoring on them.

## Literature survey

Empirical studies on code clone evolution show that evolutionary aspects of clones cannot be revealed through analysis of clones in a single version. Evolution studies are performed for different types of clones [15, 16] and for different granularity levels [17–19]. One of the earliest studies on code clone evolution in a systematic way was done by Kim et al. [2]. They performed an empirical study of clone evolution by defining clone evolution patterns in a formal notation. Based on these clone evolution patterns, clone genealogies are extracted from various versions of three software systems. Analysis of clone genealogies showed that usually clones disappeared within few check ins. This shows that aggressive refactoring of clones is not an appropriate decision for newly introduced clones. Analysis of long lived genealogies reveals that these clones cannot generally be refactored through traditional refactoring techniques. They concluded that all clones are not refactorable and hence remain in the system till the last release of the software.

Refactoring of clones is an important maintenance activity to improve code quality. A thorough literature survey was recently published on clone tracking and refactoring [20]. In the literature, refactoring of clones are studied in three different dimensions. First is clone analysis in previous versions to understand how clones are removed/refactored in a software history through refactoring techniques [21]. Second is to suggest the code clones that can be refactored through software refactoring techniques and assess the effect of refactoring on clones [22]. Third is the tool support for automatic removal of code clones. In the following, we present some studies related to these main research areas of clone refactoring.

Gode [7] studied refactoring of code clones from developers perspective. He manually analyzed how developers refactor cloned code. His main focus was on analyzing whether developers deliberately remove clones through software refactoring or the cloned code is accidentally removed i.e. during routine maintenance of software. He used source code change commits from version history to identify removed code fragments. He defined heuristics for detection of deliberate and accidental removal and also found discrepancies between cloned code and refactored code i.e. clones that are deliberately removed by developers are not reported in clone detection tools. *Extract method* refactoring is mostly used in deliberate clone removal.

An empirical study is done by Bazrafshan et al. [14] to analyze the code clone removal from developer’s perspective. They studied clone removal on eleven software systems. Their analysis concludes that most often clones are removed accidentally than deliberately. They also analyzed types of refactoring performed on removed code clones. They observed that same refactoring types are applied on code clones in both deliberate and accidental clone removals. They identified some incomplete clone refactorings i.e. developers refactored some instances of a clone class and left others. Author suggested that integration of clone management tools in development environments will help in efficient clone removal as tools will guide developers in accomplishing clone removal task on all instances of a clone class. They identified that near-miss clones are removed more often than identical clones. This observation is opposite to the conventional understanding because removing near-miss clones is considered more difficult than identical clones.

Hu et al. [23] proposed a method to determine the clone harmfulness by using the evolution history of clones. Clone harmfulness is measured through bug proneness and inconsistent changes in clones. They also identified the factors that can influence clone harmfulness and counter measures to mitigate the level of harmfulness. Analysis of five open source and three industry systems revealed that most of the clones are not harmful in terms of inconsistent maintenance overhead or bug proneness because they do not need to be maintained consistently. There are various factors that affect clone harmfulness depending upon the context of clone maintenance. Some harmfulness factors effect the cone harmfulness level such as clone classes maintained by more than one developer are more harmful than clone classes maintained by a single developer. Analysis of the clone mitigation strategies revealed that that the mitigating measures can reduce the harmfulness levels of clones. This study is different from our study because our study does not focus on harmful effects of clones, rather it analyzes how developers performed clone refactorings either consistent or inconsistent, whether they removed clones or not.

There are a variety of studies which propose automatic/semi-automatic approaches to suggest code clones that can be refactored through software refactoring techniques [9, 24–26]. Higo et al. [24] proposed a metric based method to support clone refactoring during software maintenance. They developed a characterization of code clones based upon some clone metrics to suggest clones for refactoring. They implemented the method in a tool called Aries to assist developers during clone management. Tool identifies refactoring oriented clones and also guides developer about how these clones can be refactored. For example, which refactoring technique should be applied and what will be the impact of applied refactoring technique on code. Tool classifies the clone classes into categories, that suggest what changes need to be done on these clones if a suggested refactoring technique is applied. To apply *extract method* refactoring, tool categorizes clones into four categories which suggest the type of changes (i.e. parameters added or deleted) that are required to perform this refactoring on these clones. For example, to apply ‘pull up method’ refactoring, tool calculates DCH (Dispersion of Class Hierarchy) which tells about the inheritance hierarchy among child and parent classes. This information helps developer in performing refactoring task efficiently.

Sheneamer [27] proposed an approach to automatically suggest a treatment about refactoring of clones in the system. It suggests whether a clone class needs refactoring and what type of refactoring is needed by a clone. To improve accuracy of classification results, a new method is proposed to convert clone outliers to unknown class. Bagging, K-nearest Neighbors, forestPA and decision forest algorithm are used to train clone refactoring data. Experiments on six software systems are performed and results showed that proposed approach achieved better accuracy than proposed approaches i.e. 93% and 87% on two different subject systems. Our study is different from this because it provides analysis of clones refacorings at a broader level such as it provides metrics (e.g. ratio of removed clones through refactoring), as well as version wise analysis of clone refactorings through quantitative and qualitative analysis.

Volanschi [25] presented an approach to manage clones either by clone removal through refactoring or providing clones to developers in a new look and feel so that they can decide about the clones according to their ease. They discussed the limitations of programming languages and existing clone management tools to help developers in refactoring clones. As all clones are not refactorable, providing new look and feel was helpful in deciding about clones. They developed a hybrid approach that combines refactoring and link editing to manipulate the benefits of both techniques. They also developed a prototype called Stereo based on the hybrid approach to help developers during clone maintenance. Evaluation of the hybrid approach revealed that in comparison to the existing approaches e.g. linked editors, this approach was better in expressiveness, scalability, and controllability.

Yoshida et al. [26] performed an exploratory study to find whether appropriate refactoring patterns are applied on code smells to fix them in a software. They used inFusion tool for code smell detection. They used an existing dataset of refactoring patterns applied on software entities. This dataset was detected by RefFinder tool and then manually analyzed to remove false positives. They categorize refactoring patterns for each type of code smell i.e. which refactoring type can remove a specific type of code smell. They performed experiments on three open source software systems. The results showed that corresponding refactoring patterns are not applied on code smells that is why they are rarely removed through refactoring. For example, only 26% corresponding refactoring patterns were applied in case of class level code smells and 16% corresponding refactoring were applied on method level code smells.

Choi et al. [28] proposed a clone metric based method to extract code clones as refactoring candidates. Their proposed method suggests combinations of clone metrics for extraction of code clones. They used CCFinder for clone detection along with Gemini which is a GUI of CCFinder. They used Gemini for extracting information about clone sets and clone metrics. They conducted an industrial evaluation of their proposed method using experienced industry developers. They manually evaluated the decision/results/output of developer. Results shown that combination of clone metrics are better than individual clone metrics.

Wang et al. [29] proposed a classification based approach to recommend clones appropriate for refactoring in terms of benefits, costs and risks. Training features are selected related to cloning relation in the source code, the context of clones and the evolution history of clones. A decision tree based classification algorithm is used for training the classifier model. Experiments are performed on three medium to large-sized open source projects. To measure the accuracy of results precision, recall and F-measure are used. Evaluation of the results show that their approach achieved a precision of 80% in clone recommendations for refactoring for studied system whereas precision of the recommendations for cross-project is also around 80%. High precision of the approach implied that automatic recommendations of clones for refactoring are useful for better clone management.

Yue et al. [12] proposed an automatic approach for recommending clones suitable for refactoring. They proposed a learning based approach to classify the clones into refactorable clones and non-refactorable clones. Different clone metrics were used as training features set. They extracted 34 clone features which belong to four categories such as clone code features, clone history features, relative location among clones, syntactic difference among clones and co-change features among clones. They used Naive Bayes, SOM, Adaboost, Random Forest and C4.5 for training the classification model. Experiments are performed on six open source projects. They measured accuracy of the approach using F-score within project and across projects. Their results show that accuracy of the approach was better than the state of the art approaches e.g. their F score was 83% as compared to 70%. They also identified the feature subsets that are more important for refactoring recommendations. Features belonging to the category of clone history features and co-change features contributed more towards efficient prediction of clone refactoring. Results of Adaboost are better than other learning algorithms.

AlOmar et al. [9] investigated code refactorings that are performed by the developers during software maintenance. Code refactorings are categorized into the refactorings that are performed to improve code reusability and the refactorings performed for other purposes. A refactoring miner tool is used for detecting clone refactorings in the previous versions of software system. Experiments are performed on 1828 Java systems to identify reusability related refactorings. Their results indicate that only 0.4% refactorings are performed for the purpose of improving reusability and most of them are performed on methods more frequently than classes. Their analysis revealed that reusability related refactorings impact high-level code elements e.g. methods, classes, and packages. Other types of refactorings may also impact parameters, variables and constants. This is different from our study because this study does not directly relate to clones refactoring analysis or clone evolution analysis.

All of the previous studies related to refactoring of clones either suggest clones that can be removed through refactoring or analyze clones that are removed by developers in past releases. These studies lack a longitudinal analysis on how clone classes evolve over time with perspective of clone refactorings. Some studies that analyze refactorings for code reusability are useful for assessing the intentions of developers while performing refactoring of various code smells but these studies are not directly relevant to clones refactoring analysis or clone evolution analysis. Our work perform a longitudinal study on clones in consecutive versions to investigate clone refactorings e.g. how many times a clone class is refactored during its life time, or whether a clone class is refactored regularly in multiple versions. Moreover, these studies do not investigate the types of code refactorings performed on clones during their evolution i.e. which refactoring types are performed on clones, whether all the members in a clone group are refactored simultaneously/consistently or same refactoring type is applied on them. In this study we performed in-depth analysis to explore how developers deal with clones when they perform refactoring during software evolution.

## Evolution of clones in terms of code refactorings

To investigate historical perspective of code clones in terms of refactoring between software releases, we study their evolution through clone evolution patterns. For this purpose, we propose evolution patterns for clone refactoring, define them formally and extract them in versions. In this section, we describe basic terminology for clone refactoring evolution and then we define our proposed evolution patterns for clone refactoring.

### Defining basic terminology

In this section we describe basic concepts related to clone refactoring and define code clones and clone evolution in a formal notation.

#### Clone refactoring

As we discussed earlier that the aim of code refactoring is to enhance software maintainability and code quality by removing design flaws. There are different types of refactoring that provide solutions for different types of programming problems [8]. Some refactoring techniques represent trivial refactoring that improve program comprehension e.g. *introduce explaining variable, add parameter* and *replace magic number with constant*. Some refactoring are used to improve code structure e.g. *add parameter*, *remove parameter*, *Move method* and *Inline method*. Some refactorings are used to remove duplication e.g. Replace method with method object,. ‘extract method’.

Application of refactoring on code clone fragments may remove cloning from the system and merge them into a higher abstract representation e.g. methods. Some commonly used refactoring patterns that are specifically used to remove cloning from the system are *extract method* and *pull-up method* [30].

**Fig 1:**
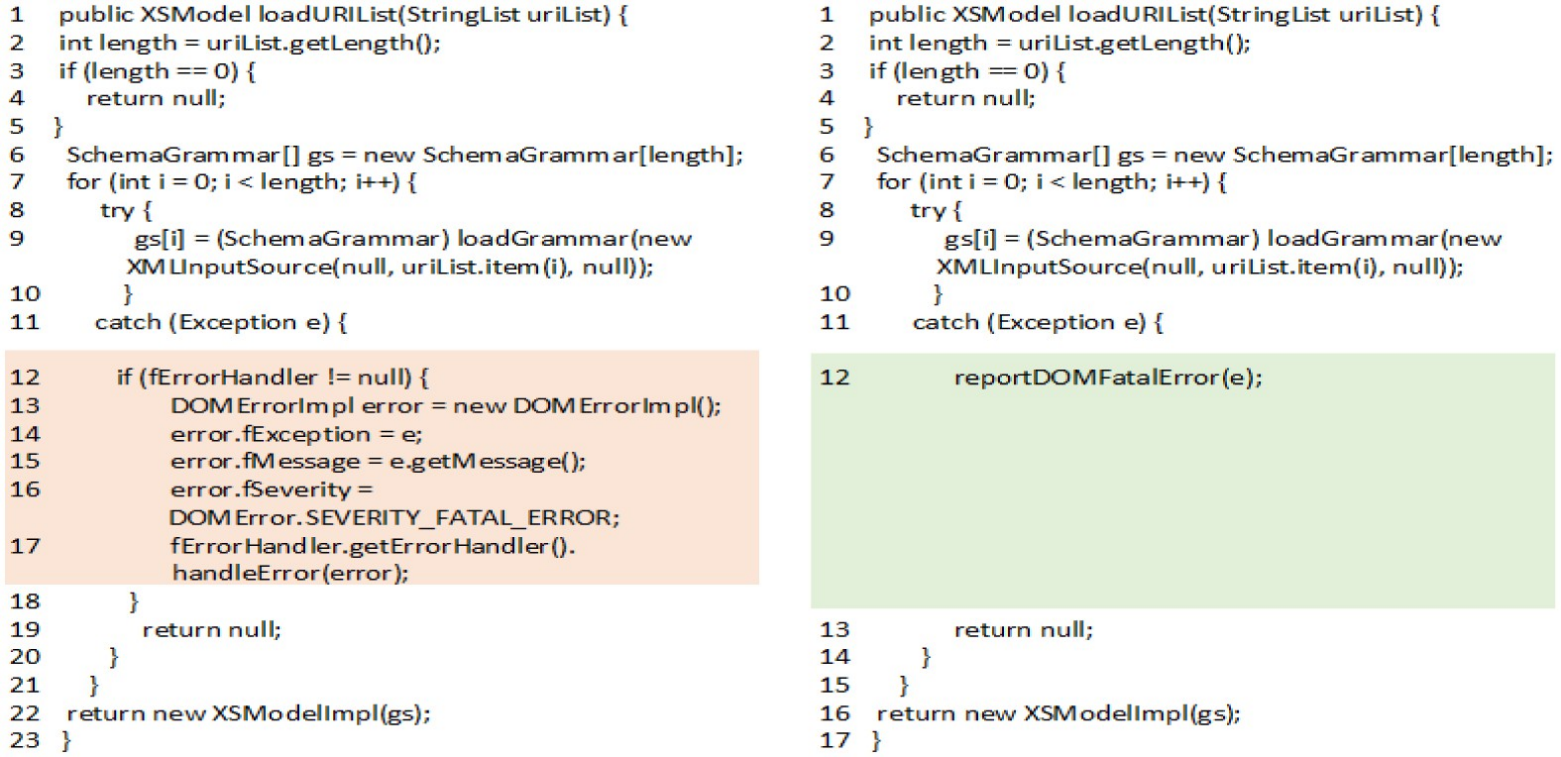
Example of clone refactoring in Xerces_J.

Refactoring techniques applied on code clones changes them differently. Fig 1 shows an example of clone refactoring where ‘*extract method*’ refactoring is applied on code fragment of a clone class from Xerces_J Version 2.6.2. The clone class consists of two clone instances reside in methods *loadURIList( )* and *loadInputList( )* of file *XMLSchemaLoader.java*. Fig 1 shows the code of method *‘loadURIList( )’* in consecutive versions i.e. Version 2.6.2 and Version 2.7.0. The cloned code contains the set of instructions for handling the error thrown by try block. Cloned code is extracted to a new method ‘*reportDOMFatalError(e)*’and is being called from original method i.e. *loadURIList( )*. Similar refactoring is performed on other code fragment of the clone class i.e. on method *loadInputList()* and clone class is removed from the software.

Before defining evolution patterns of clone refactoring, we will define basic terminology related to code clones and clone evolution in formal notation.

#### Code clones

A set of code fragments that are similar according to some similarity criteria are called clones of each other. A code fragment *cf* is a 4-tuple〈*S*, *L*, *M*, *F*〉 consisting of a chunk of source code starting at line *S* with the following *L* lines of code in a method *M* which is declared in file *F* [31].

**Clone pair.** Two code fragments *cf*_1_ and *cf*_2_ are called clones of each other if they are similar according to some similarity criteria. A *Clone pair* (*CP*) can be defined as

*CP* = {*cf*_1_, *cf*_2_} such that *sim* (*cf*_1_, *cf*_2_)>*k where k* is a similarity threshold which represents similarity of code fragments. The similarity function *sim* (*cf*_1_, *cf*_2_) can measure textual, semantic or syntactic similarity [32].

**Clone class.** Contains set of code fragments cf which are similar to each other. Each code clone fragment of a clone class is called clone instance of the class. A Clone class (*CC*) can be defined as

*CC* = {*cf*_1_, *cf*_2_, *cf*_3_, …, *cf*_*n*_} such that for any pair *cf*_*i*_, *cf*_*j*_, *sim*(*cf*_*i*_, *cf*_*j*_) > *k*, 1 ≤ *i*, *j* ≤ *n* ∧ *i* ≠ *j*. Each (cf) represents an instance of a clone class/clone pair.

**Fig 2:**
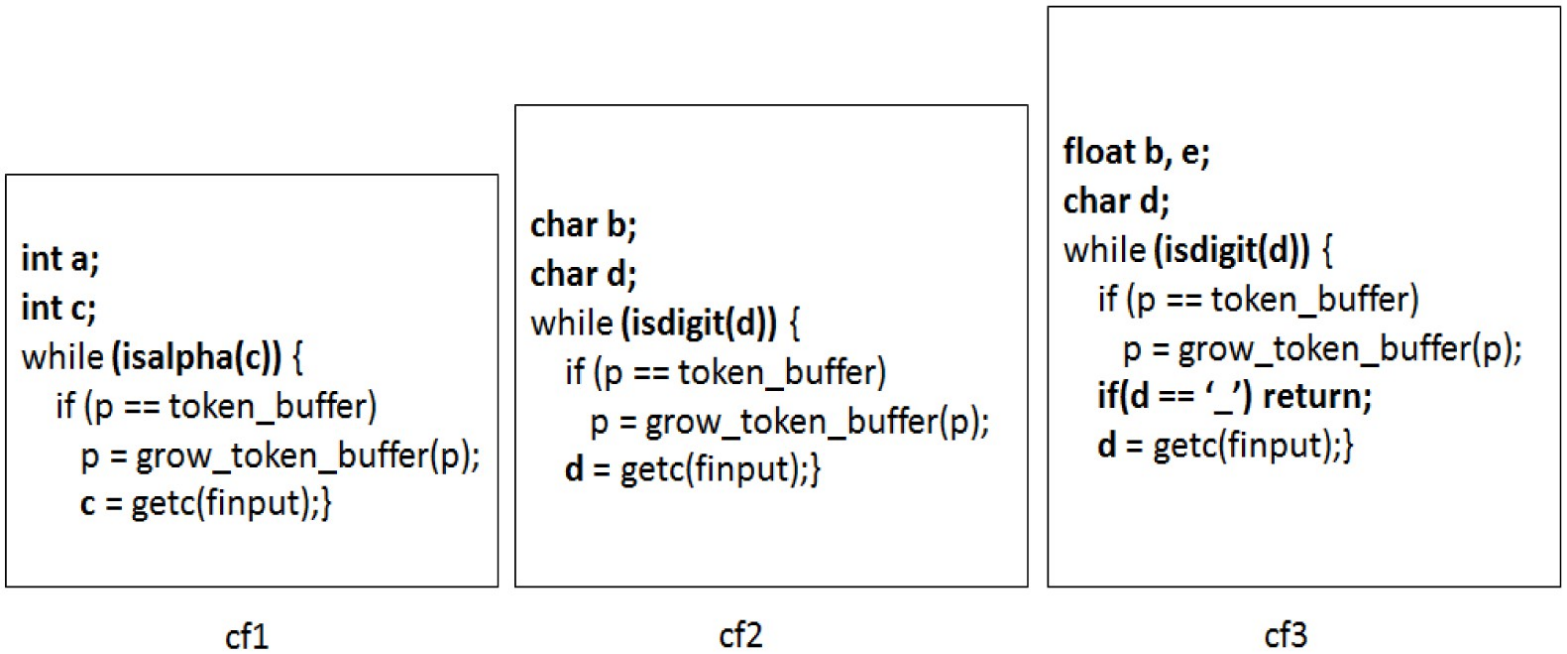
Example of code clones.

An example of code clones is shown in Fig 2 where three code fragments *cf*_1_, *cf*_2_, *cf*_3_ are similar to each other and are *clone instances* of a clone class.

We observed that code clones are refactored in variety of ways during software evolution. Developers apply different refactoring techniques on clones which sometimes remove the clones and in other cases it improves the cloned code. To understand the type of change a refactoring can make in code clones, study of clone evolution is needed.

#### Clone evolution

For the purpose of our study, a software system *S* = {*F*_1_, …, *F*_*n*_} consists of a set of *n* source code files *F*_*i*_ where 1 ≤ *i* ≤ *n*. A system exists in multiple versions *V* where the complete system can be retrieved for all versions *V*:

$$S(V)=\{FV_{1},\;FV_{2},\;\ldots ,\;FV_{n}\}$$

is the system in versions *V* where *V*_1_, *V*_2_, …, *V*_*n*_ denotes versions of a system *S*. The differences between two versions *V*_1_ and *V*_2_ of a system can be identified by a set of changes: Let *D*(*V*_1_, *V*_2_) denote the set of changes {*d*_1_, …, *d*_*k*_} between S(V1) and S(V2). Evolution of software system is the study of changes between its different versions [31].

To study clone evolution in versions, changes in individual clone class is studied in versions V of a software. For evolution analysis, software clones are detected for each version of a software and then mapped across the versions. Changes that occurred to a clone class in consecutive versions are observed to understand evolution of each clone class. A clone evolution pattern represents a kind of change that a code clone class went through from one version *V*_*n*_ to a subsequent version *V*_*n*+1_. In the literature, evolution of code clones is studied through clone evolution patterns. Refactoring of clones also represent a type of change applied on code clones. For studying clone refactoring evolution, we also define evolution patterns for refactoring. Evolution patterns for refactoring represent impact of refactoring on clones, for example, whether clones are removed in result of refactoring applied on them. It also represents how developers manage clones during software evolution e.g. whether all clone instances of a clone class are refactored in the same change task.

### Proposed evolution patterns of clone refactorings

**Fig 3:**
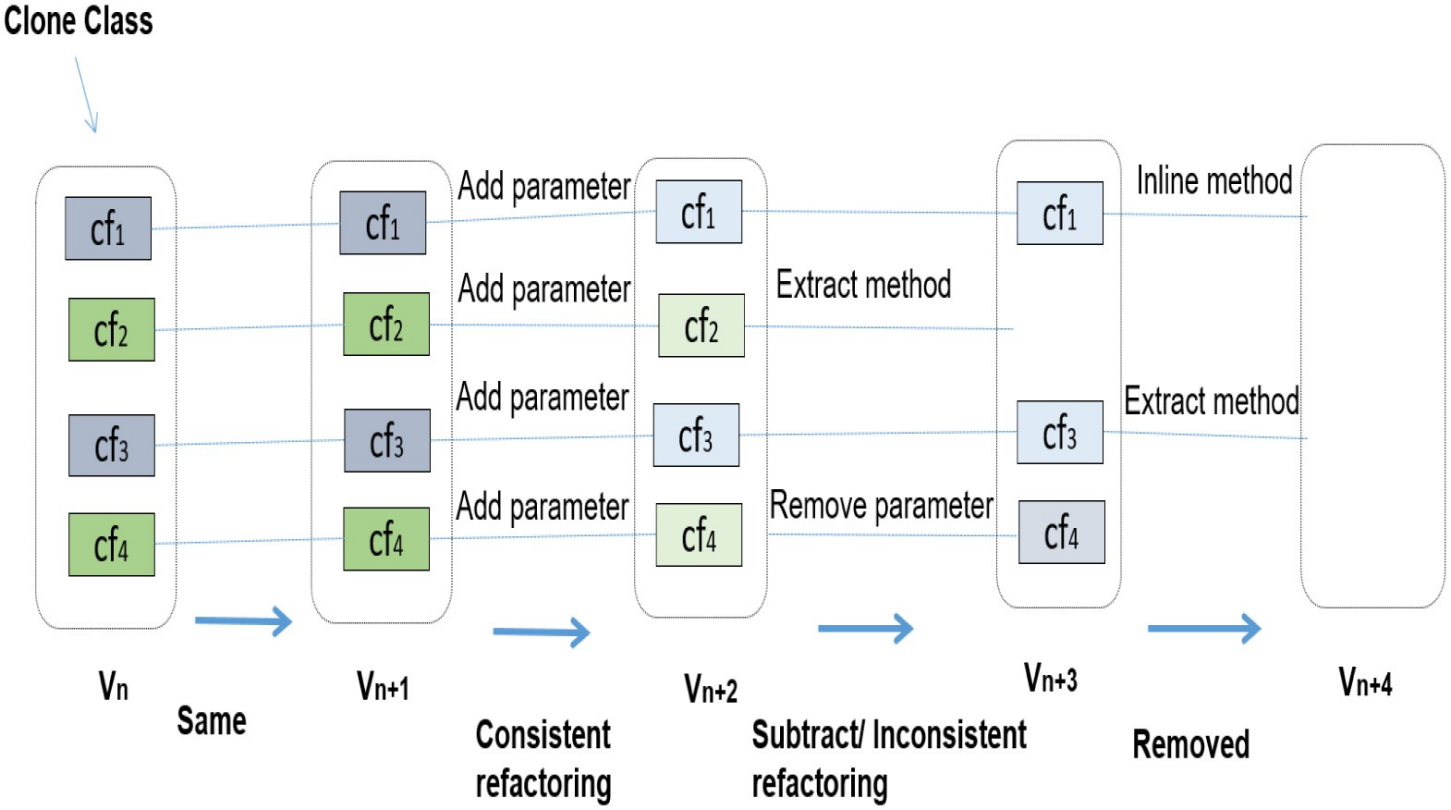
Different evolution patterns of clone refactoring between software releases.

A clone evolution pattern represents a kind of change that a code clone class went through from one version *V*_*n*_ to a subsequent version *V*_*n*+1_. Evolution patterns for code clones are defined in [2]. Following the guidelines of clone evolution patterns, we defined evolution patterns for clone refactorings. A clone refactoring pattern represents a kind of change occurred to a clone class in results of refactoring performed on its instances between the versions. Fig 3 shows some clone refactoring evolution patterns.

**Same.** None of the code fragments of a clone class in *V*_*n*+1_ was changed from *V*_*n*_. For all *cf*_*i*_ ∈ *CC*_*n*_ and all *cf*_*j*_ ∈ *CC*_*n*+1_, 1 ≤ *i* ≤ *Num*(*CC*)

$$CC_{n}.cf_{i}=CC_{n+1}.cf_{j}\;for\;all\;i=j,\;Num(CC_{n})=Num(CC_{n+1})$$

where *Num*(*CC*) represents the number of code fragments in clone class *CC*.

Fig 3 shows example of same pattern of clones where a clone class with four code fragments in *V*_*n*_ remains same in *V*_*n*+1_.

**Subtract.** At least one code fragment from *V*_*n*_ does not appear in *V*_*n*+1_ because of some refactorings applied on it.

$$CC_{n+1}=CC_{n}-\{cf_{i}\}$$

where *cf*_*i*_*isacodefragmentofCC*_*n*_, *Num*(*CC*_*n*_) ≥ *i* ≥ 1.

Fig 3 shows an example of subtract clone refactoring pattern where a clone class with four code fragments in *V*_*n*+1_ is reduced to two code fragments in *V*_*n*+2_. Two code fragments of this class disappeared because refactorings are applied on them.

**Removed.** A clone class from *V*_*n*_ does not appear in *V*_*n*+1_ because of some refactorings applied on them.

$$CC(V_{n+1})=CC(V_{n})-\{CC_{n}\}$$

Fig 3 shows an example of remove pattern where a clone class in *V*_*n*+2_ contains two code fragments. In *V*_*n*+3_, the class is disappeared because two different refactoring patterns are applied on it.

In order to define following two evolution patterns formally, we introduce the refactoring function *θ* for code fragments. *θ*: *cf* → *cf*′. It takes a code fragment *cf* and changes it to a corresponding code *cf*′ by performing some refactoring task. There can be several such refactoring tasks possible and we can denote them by *θ*_1_, *θ*_2_, …, *θ*_*n*_.

**Consistent refactoring.** All code fragments of a clone class in *V*_*n*_ appear in *V*_*n*+1_ after undergoing the same refactoring task.

$$\text{For}\;\text{all}\;cf_{i}\in CC_{n}\;\text{and}\;\text{all}\;cf_{j}\in CC_{n+1},1\leq i,j\leq size(CC)$$

$$CC_{n}.cf_{i}=CC_{n+1}.cf_{j}^{\prime }\wedge Num(CC_{n})=Num(CC_{n+1})$$

where *θ*(*cf*) = *cf*′ represents a refactoring task on code fragments.

Fig 3 shows an example of consistent refactoring of clones where a clone class contains four code fragments in *V*_*n*_. Same refactoring pattern (add_parameter) is applied on all the four code fragments of this clone class. Consistent clone refactoring represents that developers are aware of clone entities in the software. That is why we can call consistent refactoring as clone-aware refactoring.

**Inconsistent refactoring.** At least one code fragment from *V*_*n*_ was refactored differently than others in *V*_*n*+1_. Different code fragments can undergo different types of refactoring.

$$CC_{n}.cf_{i}=CC_{n+1}.cf_{j}^{\prime }\;\wedge \;CC_{n}.cf_{i}=CC_{n+1}.cf_{j}^{\prime \prime }$$

where *θ*_1_(*cf*) = *cf*′∧*θ*_2_(*cf*) = *cf*′′ and so on.

Fig 3 shows inconsistent clone refactoring where a clone class contains four instances in *V*_*n*+1_. Two different refactoring patterns (remove_parameter and extract_method) are applied on only two clone instances of this clone class. This represents that developers may not be aware of cloning in the system or clone instances of a clone class require different types of improvements in their code.

## Experimental setup

**Fig 4:**
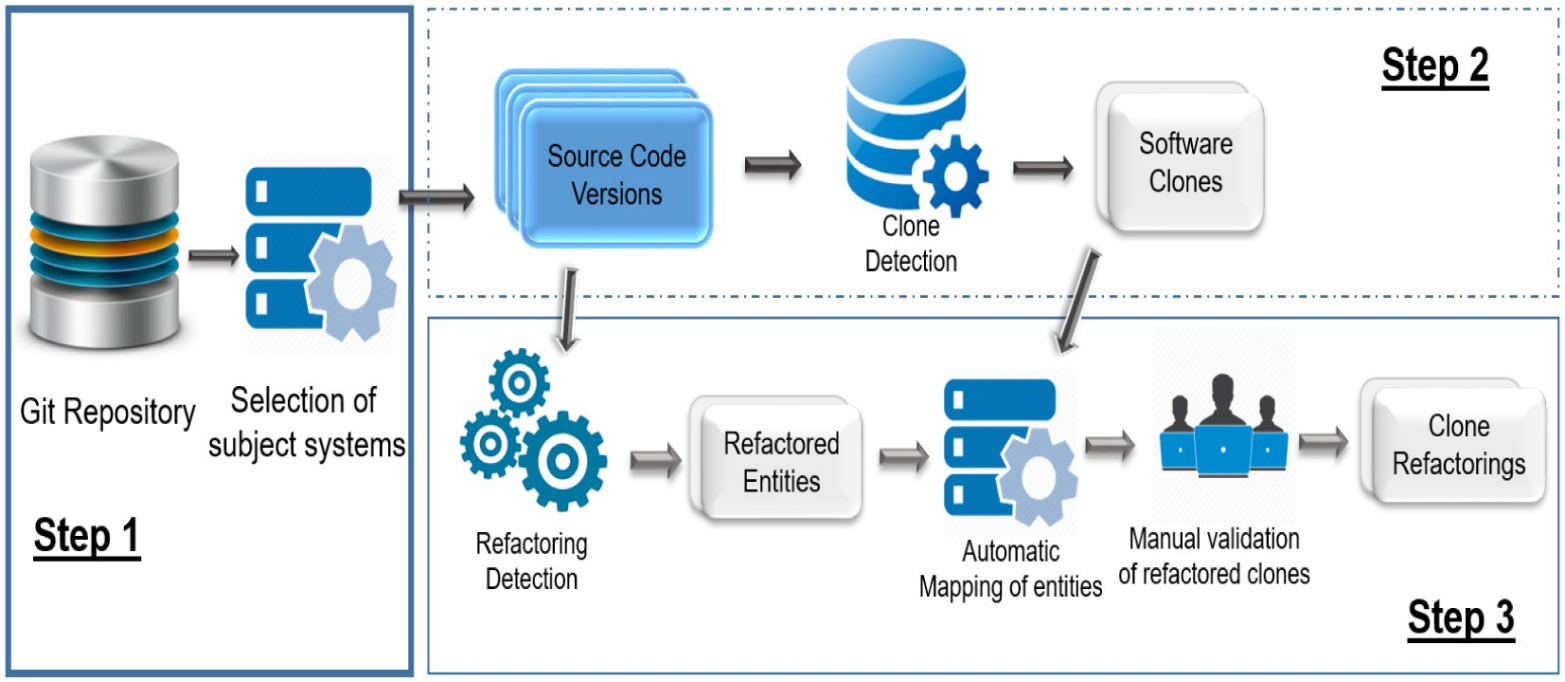
Experimental steps for clone refactoring evolution.

In this section we describe the detailed setup for our study. Fig 4 shows various steps to perform clone refactoring analysis in software versions.

In the first step, we selected subject systems from GIT repository [33]. To perform the study of clone evolution, software systems are selected that contain an evolving history (multiple versions released over a period of time) because such systems are consistently enhanced in terms of code quality and software features. As these systems are open source, these are developed and maintained by multiple developers which reduces the biasness in terms of programming style (e.g. copy paste activity) which may occur in case of a single developer [34–36]. Our selected systems are well known systems and have been studied previously for clone research. We selected five Java systems to conduct experiments i.e. JabRef, Xerces_J, Guava, JFreeChart and JHotDraw. Table 1 shows the information about the selected versions of these systems.

**Table 1:** Statistics of software systems.

Subject Systems	# of releases	Start date	End date	Starting release	# of methods
Jabref	13	Feb, 2015	Aug, 2016	2.1	4678
Xerces_J	14	Jan, 2003	Nov, 2010	2.3	5897
Guava	13	Oct, 2011	Dec, 2015	10	8998
JFreeChart	13	Dec, 2006	Nov, 2017	1.0.8	8526
JHotDraw	11	Feb, 2001	Jan, 2011	5.2	6344

JabRef is an open source reference management system that uses BibTEX and BibLATEX to provide graphical interface for the LATEX typesetting system [37]. Xerces_J is Apache’s collection of software libraries that uses various standard APIs for parsing, validating and manipulating XML schema [38]. Guava is a set of Java libraries containing core utility functions for providing features like functional programming, graphs and caching [39]. JFreeChart is an open source Java chart library that provides user to make high quality charts for their applications [40]. JHotDraw is a Java framework for technical and structured drawing editors. It is very well known for software design patterns [33]. We selected some of the latest versions of these systems. Details of these software systems are given in Table 1.

### Clone detection

To study clones in versions, we need to detect code clones for each version of software. For this purpose, we used the SoucererCC [41] tool. SoucererCC is a token based clone detection tool. We set the minimum similarity threshold of 50 tokens for clone detection as suggested in the literature [41]. SourcererCC detects clones at different granularity level e.g. code fragment level, method level, file level. We selected code fragment level clones because most of the refactorings are performed at code fragment levels such as *consolidate duplicate conditional fragments, replace method with method object* and *extract method*.

### Refactoring detection

In order to gain knowledge of refactoring performed between two versions, we ran Ref-Finder [42] tool on the consecutive versions of software. Ref-Finder tool is an Eclipse plug-in that takes two program versions as input and extracts well-known refactoring patterns [8] from them. Ref-Finder tool is used in different refactoring studies [26]. Ref-Finder reports refactoring information that helps in detecting what type of code refactoring is performed between the two versions, where it is performed (e.g. method name, file name and file path). It also provides some information about what changes occurred in next version as a result of these refactoring. This information helps in identifying exact lines of code where refactoring is performed and thus in mapping clone code fragments with refactored entities. In the literature, it is reported that RefFinder may produce false positives [43]. In order to counter this, a manual analysis is performed for each refactoring to remove false positives. Detail of manual analysis is given in the next section.

### Clone refactorings extraction

To identify clone refactoring, we need to map refactoring with code clones. Refactorings detected by Ref-Finder tool are reported at method level, class level or interface level, referred to as refactored entities in this paper. The methods and classes where clones reside are referred to as clone entities. As Ref-Finder tool report refactoring results at method level, we first mapped clone entities with refactored entities at method level. Our tool *CRefEvol* maps clone entities with refactored entities. It takes two files as input: One is the refactoring file that contains all the code refactorings performed on a software system between two versions e.g. *V*_*n*_ and *V*_*n*+1_. This file contains location of code where refactoring is performed (e.g. method name and file/class name) and type of refactoring performed on it. The other file is the code clone file of the version *V*_*n*_ on which refactorings are performed. Method names and file names of each instance of a clone class are matched with the refactoring file. If any instance of a clone class is matched with the refactored code entity we consider them as refactored code clone fragments *cf*′. Clone classes that contain the *cf*′ are called refactored clone classes.

### Validation of clone refactorings

After locating the refactored clone entities, we performed manual analysis on them to remove the false positive refactorings reported by RefFinder. The manual validation of RefFinder results is performed after mapping the refactorings with clones to reduce the effort of manually analyzing all refactorings reported by the tool in a software. Manual validation is performed by three experts. They are all Computer Science graduates, two of them have experience of software development of more than three years and one is a research student. The experts are asked to answer the following two questions:

(1) Is reported refactoring a true positive?
(2) Is reported refactoring performed on the mentioned location in the file?

For manual validation, we gave the results of mapped clone refactorings for the five subject systems to the experts. The mapped refactorings contain the information of lines of source code file of clones in files of consecutive versions, type of refactoring performed on them. As a result of manual validation, false positives are removed by experts and the mentioned location of the reported refactoring was also verified.

### Detection of evolution patterns of clone refactorings

To detect evolution patterns of clone refactoring, we need to trace each refactored clone class of a version *CC*_*n*_ to clone classes of next version *CC*_*n*+1_. Our tool CRefEvol detects the evolution patterns defined in section of ‘Proposed Evolution Patterns of Clone Refactorings’ and uses CRD (Clone Region Description) method [44] to map clones between two versions. In this method, instead of source code, region of code is mapped. To identify different evolution patterns, we perform mapping at the clone instance level between clone class of consecutive versions. For example to identify removed clone classes, we matched the clone instances of a class to the clone instances of the next version. If all the instances of a clone class of a version disappeared in the next version we consider it as removed clone class. To identify subtract clone refactoring, if some of the instances of a refactored clone class disappeared in next version, it is considered as subtract pattern. Subtract pattern represents removed clone instances as a result of refactoring.

### CRefEvol tool IDE

**Fig 5:**
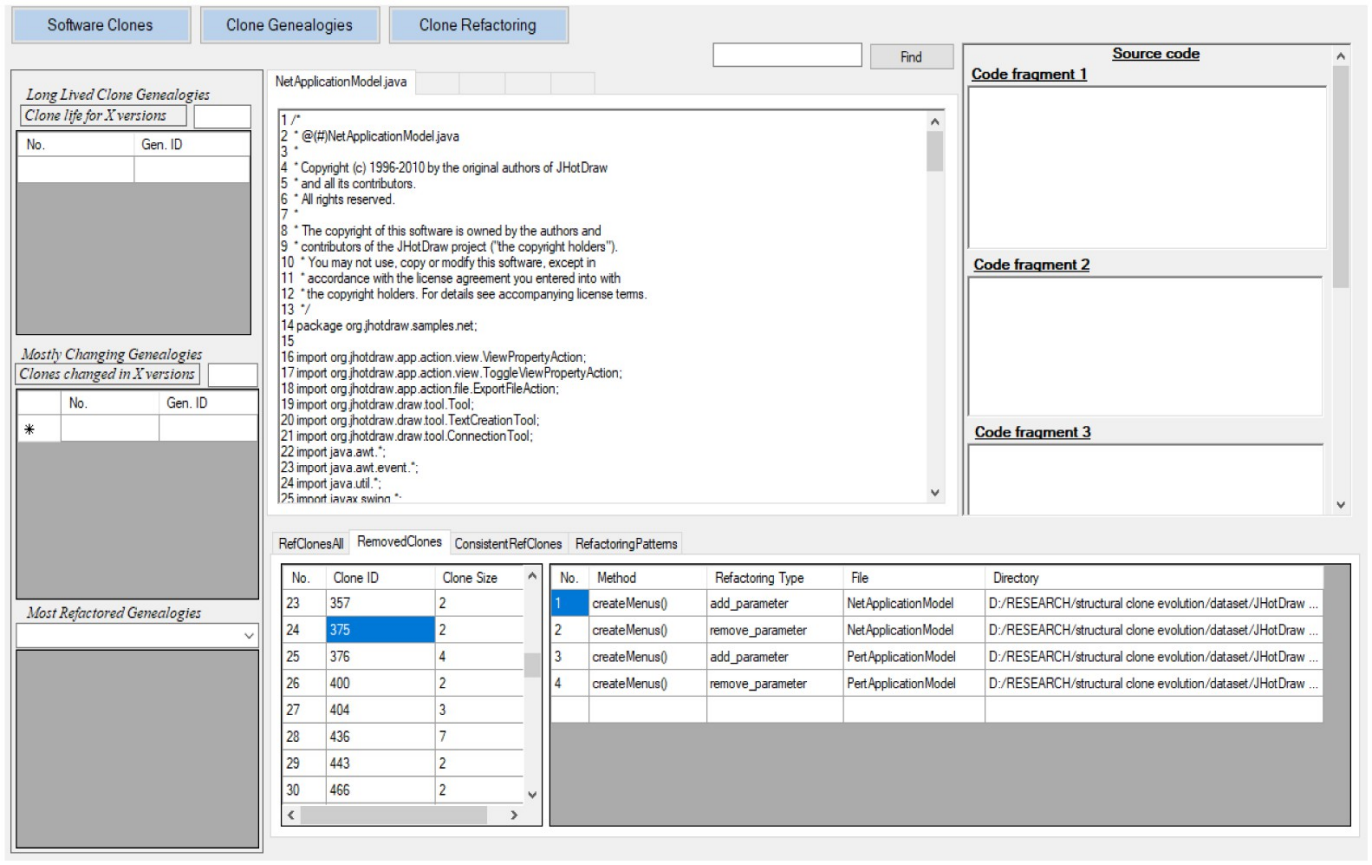
Screenshot of CRefEvol tool, RefactoredClones view.

Our tool CRefEvol represents the clone refactorings performed in a system as shown in Fig 5. It displays all the refactorings performed on the clones between any two versions. It displays clone ID and clone class size for each refactored clone class in a table. By clicking on the clone ID, it displays method and file name of clone instances of a clone class, refactoring patterns applied on each clone instance and directory (file path) of that file. By clicking on the directory it opens the file in a text editor. User can search the relevant method in the file. Information of all clone classes that are removed as a result of refactoring and information of clone classes that are consistently refactored are also shown in tables. In another tab, frequently applied refactoring patterns are shown and frequency of each pattern is also shown.

## Results and analysis

In this section, we discuss the results of clone refactoring in different versions of five Java systems. We first present the clone statistics of the five Java software systems. Table 2 shows the number of clone classes, average number of clone instances, average number of instances per clone class and their standard deviation in a clone class for five Java systems. These averages are calculated in different versions of these systems. Table 2 shows the averages in min—max format which represent minimum and maximum averages in different versions e.g. 1227—1375 represents that the minimum average number of clone classes is 1227 and the maximum average number of clone classes is 1375 in studied versions of Jabref.

**Table 2:** Statistics of clone refactorings in five java systems.

Software Systems	Avg. # of clone classes	Avg. no. of clone instances	Avg. # of instances per clone class / standard deviation	Avg. release time (days)
Jabref	1227–1375	3056–3982	2.7/ 1.8	129
Xerces_J	1180–1686	3025–4432	2.5 / 1.3	116
Guava	1045–1137	2965–3287	2.9 / 1.6	34
JFreeChart	1324–1528	5707–6768	4.3 / 5.5	309
JHotDraw	13–1335	42–3700	2.7 / 1.5	96

To analyze clone refactorings in versions, CRefEvol provides different quantitative measures (e.g. *percentage of refactored clones*, *percentage of removed clones*) and also visualizes evolution of individual clone classes across versions of software that helps in qualitative analysis. We analyze evolution of clones refactoring by answering the following questions.

*RQ1: What ratio of code clones are refactored between the releases in software systems?*

*Major findings:* A small proportion of clones is refactored between consecutive versions. Developers apply the refactoring patterns that improve the quality of cloned code instead of the ones that remove clones.

### Quantitative analysis

Table 3 represents the *percentage of refactored clones* in each version of five Java systems. A high ratio of refactored clones (12.3% and 11.89%) is observed in JHotDraw version 7.1 and version 7.5 respectively. Refactoring ratio remains less than 1% in most of the versions of JabRef, Guava and JFreeChart. On average, the number of refactored clones (*cf*′) in different versions in these five systems ranges from 0 to 440 (12%). refactoring ratio in JHotDraw (7%) is higher than other systems and in JFreeChart, it is lower than other systems e.g. on average 0.26% clones are refactored. This shows that on average, a very small proportion of code clones are refactored between consecutive versions.

**Table 3:** Percentage of clone refactoring ratio in versions of five Java systems.

Versions	Jabref	Xerces_J	Guava	JFreeChart	JHotDraw
Vn	0.23	1.79	0.42	0.36	11
Vn+1	0.03	5.12	0.31	0.3	9.09
Vn+2	0.03	5.99	0.46	0.09	8.66
Vn+3	0.03	1.76	0.44	0.53	4.07
Vn+4	0.54	1.06	0.56	0.62	9.76
Vn+5	3.34	6.1	3.65	0.21	12.33
Vn+6	1.02	0.05	9.24	0.09	3.25
Vn+7	1.69	1.28	0.67	0.24	7.19
Vn+8	1.04	0.82	0.39	0.09	4.32
Vn+9	0.23	0.87	0.66	0.02	11.89
Vn+10	0.64	1.33	5.55	–	–
Vn+11	–	4.8	7.53	–	–
Vn+12	–	0.45	–	–	–
Average	0.78	2.47	2.49	0.26	7.84

### Qualitative analysis

**Size of refactored clone classes.** Analysis of clone class size that are refactored during evolution will indicate the relation of clone class size with clone refactoring. For example, whether developers performed refactoring on small/large clones during software evolution. For clone class size, we analyzed it for Num(CC) (number of instances in a clone class) and LOC of clone class. Num(CC) of refactored clone classes and non-refactored clone classes is almost same. For example, for Xerces_J, average Num(CC) is 2.4 for refactored clones and 2.6 for non-refactored clones (smallest among all systems) and for JFreeChart, average Num(CC) is 4.4 for refactored clones and 4.3 for non-refactored clones. Analysis of LOC of clone classes shows that clones with larger source code fragments are refactored more often than clones with smaller code fragments. Clones with smaller code fragments may be simple code which require less improvements as compared to large code fragments. Most of the refactoring types are applied on large code fragments e.g. ‘*Move method*’, *extract method* and *consolidate duplicate conditional fragments* are applied on large code fragments. This analysis will guide developers in giving high priority to large code clone fragments while prioritizing clone refactoring tasks.

**Refactoring patterns.** Identification of refactoring patterns commonly used in clone refactoring tasks in previous versions will indicate developer’s approach for refactoring clones. We analyze clone refactorings to see which refactoring patterns are mostly applied on code clones in our subject systems. Most common refactoring patterns performed on code clones in versions of five Java systems are *add parameter*,*remove parameter*, *introduce explaining variable* and *replace magic number with constant*. These represent trivial refactoring types that improve program comprehension. Other common patterns include *Replace method with method object, replace nested conditional guard classes*, *Move method*, *extract method* and *consolidate duplicate conditional fragments*. These results indicate that most of the refactoring techniques applied on our studied systems are the refactorings whose purpose is to improve program comprehension and code structures. Refactoring techniques that remove cloning from the system such as *Pull up method* and *extract method* are less commonly used than other types. This shows that generally, developers perform clone refactoring in a software system to improve the quality of cloned code instead of removing them from the system. The developer’s behaviour for performing clone refactoring will guide researchers and practitioners while devising tools for code clone refactoring.

**Fig 6:**
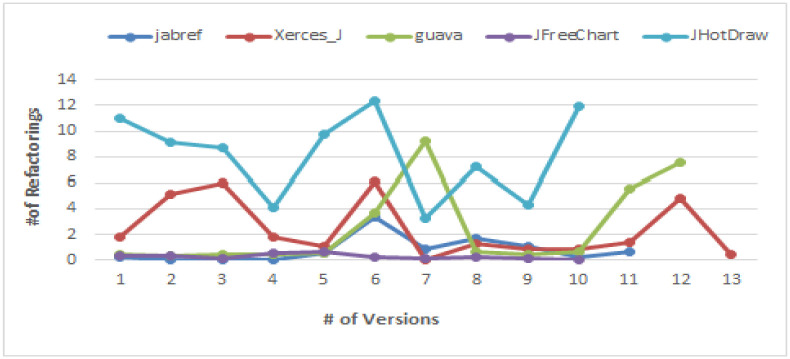
Clone refactoring ratio in versions of five Java systems.

**Version wise analysis of clone refactoring ratio.** Analysis of clone refactoring in various versions will help in understanding the management approach for refactoring clones in versions. For example, whether clone refactoring is a regular activity which is performed in all the versions with same ratio or clone refactoring ratio changes in different releases/requirements. Fig 6 shows the graph of clone refactoring in various versions of five Java systems. There is no continuous increasing or decreasing trend in versions for all the five systems. Number of refactored clones varies in different versions. For example, in Xerces_J, there is a great variation in refactored clones in some versions. In version 2.6.2 (Vn+5), refactored clones are 219 (6.1%) but in next version, only two clone refactoring are performed. In Guava, in version 14.0.1 (Vn+6), refactored clones are 300 (9.2%) but in next version they are 20 (0.6%). This shows that number of clone refactoring performed in versions varies widely.

**Fig 7:**
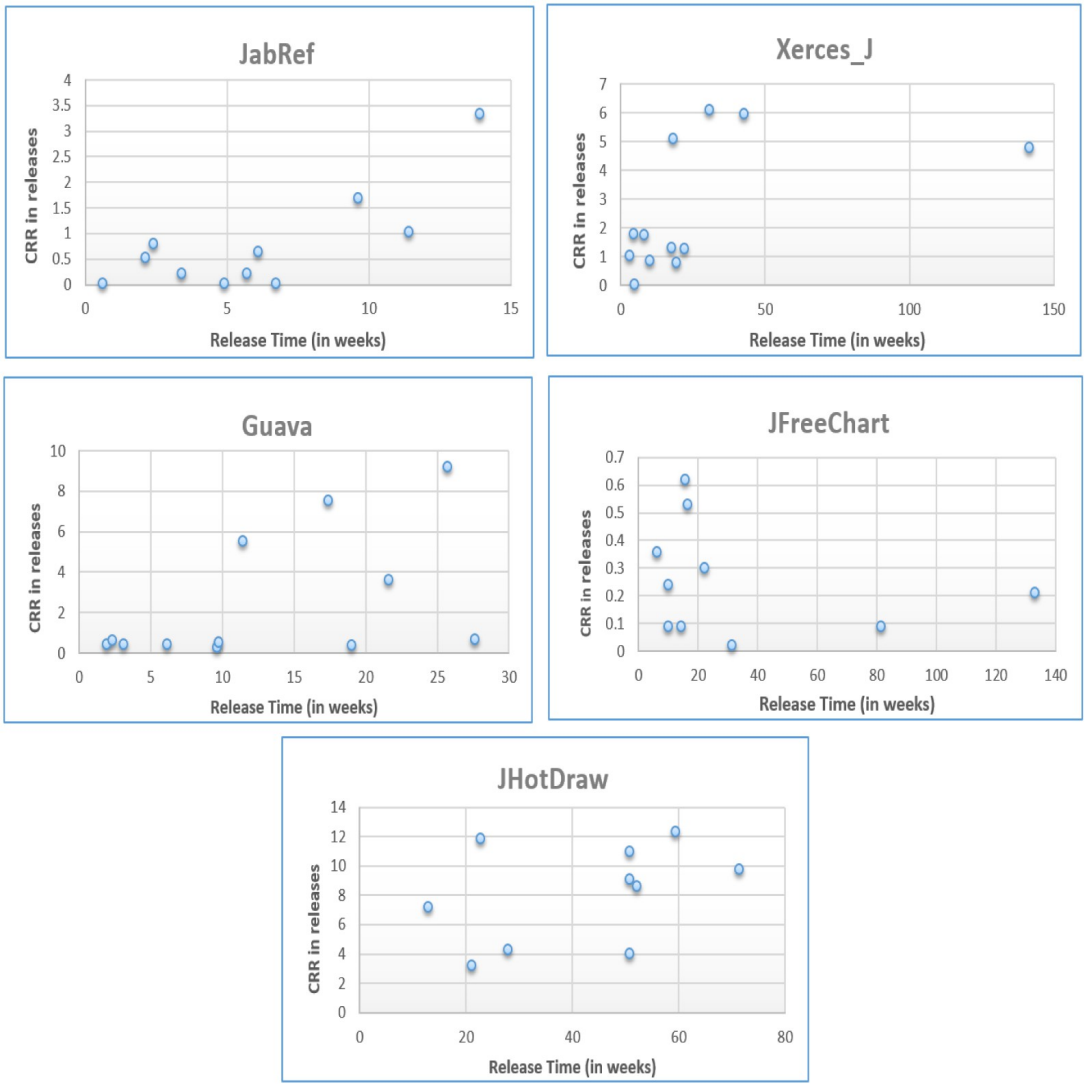
Clone refactoring ratio and release time duration for five Java systems.

Fig 7 shows the clone refactoring ratio and release time duration (in # weeks) in various versions of five Java systems. For different systems, release time duration varies widely e.g. maximum releases time of JFreeChart is 132 weeks (with an average of 34 weeks) and maximum release time of JabRef is 14 weeks (with an average of 5 weeks).

Analysis of release time in versions shows that generally clone refactoring ratio is greater in larger release time duration. For example, in JHotDraw, there are five releases with time duration more than 50 weeks and clone refactoring ratio is greater than 8% and in Guava, there are three releases with time duration more than 10 weeks and clone refactoring ratio is greater than 5%. This is because developers generally perform refactoring when they have more time between the releases.

There are some releases where release time is short (Xerces_J V_2.7, JHotDraw V_7.2) but clone refactoring ratio is greater. In this case, most of the time the release type is a major release. The reason may be the need of more efficient software in terms of code quality to satisfy users expectations from major releases.

*RQ2: Does code refactoring remove clones from the system?*

*Major findings:* 33% of total refactorings remove clones in all the studied systems. Smaller clone classes (Num(CC)) are removed more often. Major causes of clone removal through refactorings include: (1) type of refactoring pattern applied on clones (2) inconsistent refactorings applied frequently on a clone class.

#### Quantitative analysis

To analyze removed clones, CRefEvol measures the percentage of removed clones over total refactored clones in a system. Table 4 shows the percentage of removed clones in various versions of five Java systems. The results show that in different versions, the percentage of removed clones is different. In most of the versions, it is less than 30% which indicates that most of the clones are not removed between the releases. There are some versions where percentage of removed clones is greater that 50% e.g. JHotDraw, *V*_*n*+9_ (65 clone classes are removed), Xerces_J, *V*_*n*+10_ and Guava *V*_*n*+11_. Analysis of the release history of these versions reveal that these are mostly later versions of the software systems where software need to be stable and developers may take decisions about removing cloning where possible, specially where it may be better for system evolution in the future. Further analysis of these versions shows that some of these versions are major releases also. For example, Xerces_J, *V*2.9 (*V*_*n*+10_) was a major release where DOM Level 3 serialization support of Xerces was migrated to a common serialization codebase shared by Xerces_J and Xalan (an XSLT processor for transforming XML documents into other document types). This shows that in major releases, developers perform refactoring that remove clones from the systems.

**Table 4:** Removed clones in versions of five Java systems.

Versions	Jabref	Xerces_J	Guava	JFreeChart	JHotDraw
Vn	33.40%	5%	23%	13.70%	50%
Vn+1	23.60%	8%	22.32%	2.70%	33.33%
Vn+2	29.40%	22.22%	30.65%	7.70%	50%
Vn+3	15.40%	17.65%	0%	5.70%	0%
Vn+4	10.40%	5.88%	5%	16.70%	0%
Vn+5	35.40%	9.88%	15%	4.76%	29.51%
Vn+6	16.67%	0%	32.00%	16.67%	28.89%
Vn+7	54.88%	5.56%	38.36%	12.50%	55.59%
Vn+8	54.25%	0%	50%	12.50%	24.24%
Vn+9	33.33%	10.53%	50%	20%	57.56%
Vn+10	28.57%	55.86%	0%		
Vn+11		12%	51.35%		
Vn+12		11%			
Average	30%	13%	26%	11%	33%

On average, percentage of removed clones ranges from 11% to 33% for the five Java systems. This shows that refactoring helps in removing a small proportion of code clone classes during software evolution. The purpose of most of refactoring types is to improve the structure of software system which may not cause removal of code clones.

#### Qualitative analysis

*Size of removed clone classes.* We analyzed the size of removed clone classes to understand the relation of clone class size (NUM(CC)) to clone removal. Our results show that removed clone classes are smaller in size than other refactored clone classes. For example, average NUM(CC) of removed clones is 1.9 (Xerces_J) to 2.4 (JFreeChart) whereas average NUM(CC) of alive refactored clones is 2.8 (JabRef) to 6.1 (JFreeChart). This shows that larger clone classes are refactored more often than smaller clone classes but not removed. Smaller clone classes are easy to remove such as if a clone class contains two clone instances, refactoring applied on only one instance can change its code from other clone fragment of the clone class and thus they may not remain clones of each other. It may be the case that large clone classes are the result of some design decision and they may not be removed by applying existing approaches whereas small clone classes are created on temporary basis to add new functionality and then cloning is removed by applying some refactoring techniques. This shows that overall, small clone classes are more frequently removed than large clone classes.

**Fig 8:**
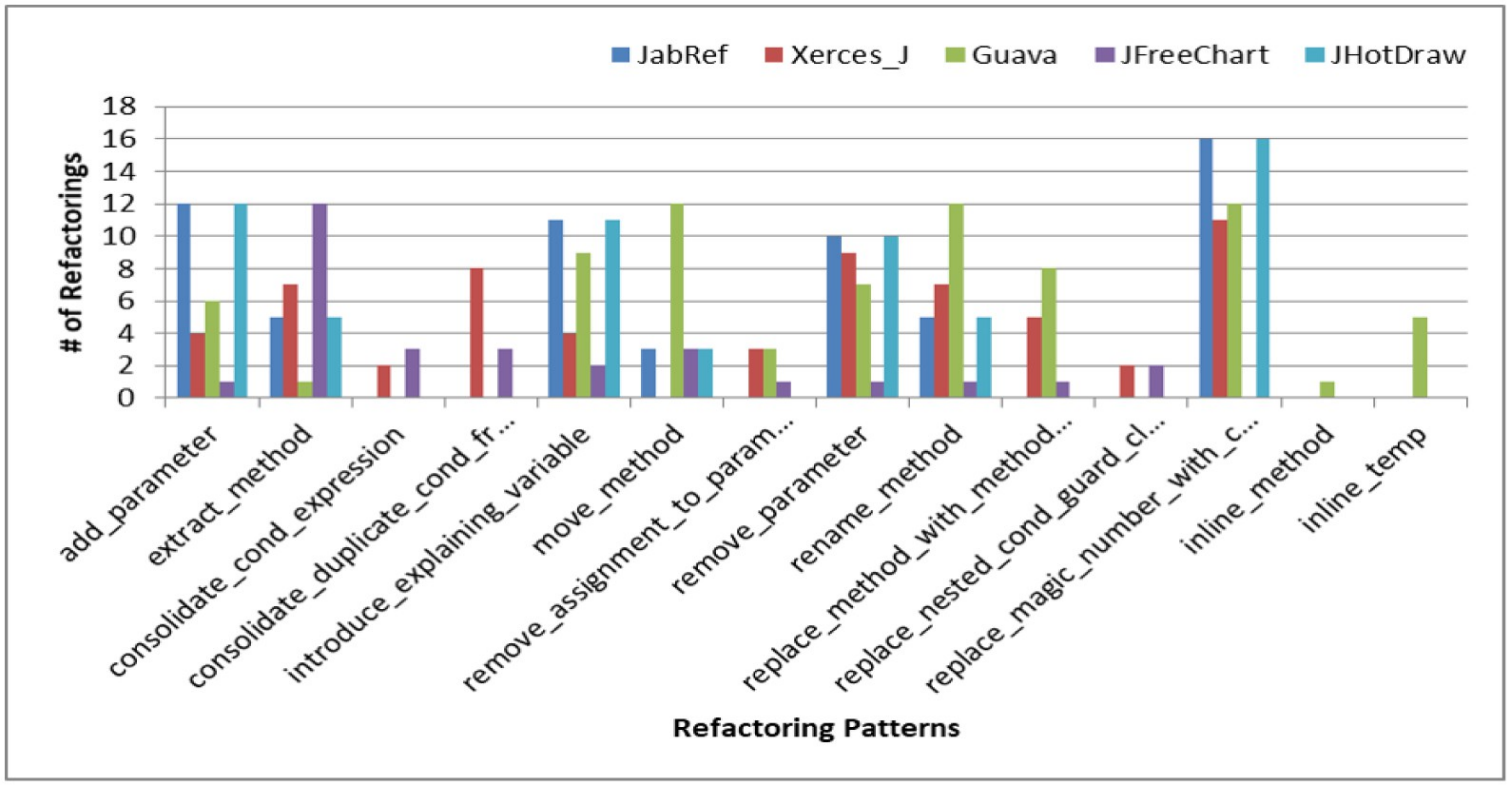
Refactoring patterns in removed clones.

*Refactoring types in removed code clones.* We analyzed clone refactorings to understand causes of clone removal. Selection of refactoring pattern for clones indicates a developer’s intention such as whether a developer wants to remove clone or he is performing refactoring for code improvement. For removed clones we analyze which refactoring types are mostly used in different versions. Fig 8 represents the average number of times the various refactoring patterns are used to remove code clones in different versions of five Java systems. The commonly used patterns in all systems are *add parameter*, *extract method, introduce explaining variable, move_method, remove parameter* and *add parameter*. From these patterns, only *extract method* refactoring is a refactoring type whose sole purpose is to remove clones from the system whereas the purpose of other patterns is to improve the structure of software e.g. *add parameter, remove parameter*, or to improve its comprehensibility e.g. *introduce explaining variable, add parameter*. To investigate the reason of clone removal, we manually analyze the refactoring performed on removed clones. Our observations reveal two main reasons of clone removal. In the first case, clone classes are removed when clone removal refactoring are applied once on them such as *extract method* or *Pull up method* is applied on clone instances. Another reason of clone removal is frequent and inconsistent refactoring i.e. some clone instances are refactored more than once either with the same refactoring pattern or with different refactoring patterns. For example, in some cases a clone class is removed because *add_parameter* refactoring pattern is applied more than once on same clone instance. In other cases, more than one refactoring are applied on same clone instances. Thus sometimes it is not a single refactoring pattern that causes the clone removal but a number of trivial refactoring are applied on a clone class which causes its removal. This shows that in most of the cases of clone removal, developers are not intentionally removing clones. The main reason of clone removal in most of the cases is independent evolution of code clone fragments of a clone class which indicates that generally, clones are removed unintentionally.

*RQ3: Are clones refactored consistently/inconsistently during software evolution?*

*Major findings:* Number of consistent clone refactorings lie in the range of 10% to 30% for the studied systems.

We perform quantitative and qualitative analysis for consistent clone refactoring to understand how maintainers deal with cloning in the system.

#### Quantitative analysis

To determine the consistency in clone refactoring, we analyzed the consistent and inconsistent clone refactorings in versions of five Java systems. As described in section of Proposed Evolution Patterns of Clone Refactorings, a consistent refactoring represents that same refactoring pattern is applied on all clone instances whereas in an inconsistent refactoring either clone classes are partially refactored (some clone instances of a clone class are refactored while others left non-refactored), or different refactoring patterns are applied on different code fragments of a clone class. CRefEvol measures the percentages of consistent and inconsistent clone refactorings in software. Table 5 shows the percentage of consistent clone refactorings in consecutive versions for five Java systems. Highest number of consistent refactorings (42%) are performed in JabRef in Version 3.0. In some versions, number of consistent clone refactorings is zero e.g. Guava Version 17.0 which indicates that all clone refactorings performed in these versions are inconsistent. In JHotDraw, consistent clone refactorings is more than 20% in all versions. On average the highest number of consistent clone refactorings is 30.5% in JHotDraw and lowest is 6.5% in JFreeChart. Overall, the consistent clone refactorings lies in the range of 10% to 30% which indicates that most of the refactorings are inconsistent in different versions of studied systems.

**Table 5:** Consistent refactored clones in versions of five Java systems.

Versions	Jabref	Xerces_J	Guava	JFreeChart	JHotDraw
Vn	17.15	8.26	18.57	5.42	27.78
Vn+1	21.88	16.36	27.12	7.08	37.04
Vn+2	27.78	19.48	15.04	5.95	27.78
Vn+3	15	19.28	18.05	9.58	27.78
Vn+4	9.09	19.89	23.03	4.17	20.52
Vn+5	42.31	20.45	29.24	12.5	29.63
Vn+6	17.65	0	11.44	7.58	20.2
Vn+7	25.76	15.59	21.2	5.95	37.04
Vn+8	14.29	19.89	7.52	5.95	22.57
Vn+9	10	25.25	7.52	0	27.3
Vn+10	11.54	9.88	0	8.75	
Vn+11	12.5	6.5	20.47	5	
Vn+12		19.55			
Average	18.75	15.41	16.6	6.5	30.54

#### Qualitative analysis

We investigate inconsistent refactoring to analyze how refactorings are applied and what are the reasons of inconsistencies. Our analysis shows that in some cases all clone instances of a clone class are refactored but different refactoring types are applied on them. This shows that developers may know about cloning in this case but clone instances of a clone class require different types of improvements. In some cases, some of the clone instances of a clone class are refactored frequently as many trivial refactoring are applied on them. In this type of inconsistent refactoring, the refactored clone fragments are removed (subtracted) from the clone class as many trivial refactoring changed their source code from other code fragments of a clone class. This type of removal is called *subtract* refactoring as discussed in section of Proposed Evolution Patterns of Clone Refactorings. An example of this type of refactoring is discussed in case 1 in the following section.

In the following we discuss some examples of consistent and inconsistent refactoring performed on code clones to understand the reasons of their consistency/inconsistency.

#### Case 1: Example of inconsistent refactoring

In Guava version 15.0, there are two clone instances in methods dispatch( ) and executeListener( ) of files EventBus.java ExecutionList.java respectively. Add-parameter and remove-parameter refactoring are applied twice on clone instance of dispatch( ) method as shown in Table 6 whereas no refactoring is performed on the clone instance of executeListener( ) method. Reason of inconsistent refactoring is the independent evolution of the methods i.e. dispatch() method requires the refactorings whereas executeListener( ) does not require these refactorings. Four refactorings applied on the dispatch ( ) method changed its source code from source code of executeListener( ) and clone class is removed. Analysis of source code of this clone class reveals that code size of the code fragments of this clone class is small. That is why trivial refactoring performed on one code fragment reduced the size of matched code with other clone instance which causes clone removal. This represents that clones with small code size may be removed by applying trivial refactoring.

**Table 6:** Example of inconsistent clone refactoring in Guava.

Method name	File name	Refactoring patterns	Location
dispatch( )	/eventbus/EventBus.java	*add parameter*	EventSubscriber:subscriber
dispatch( )	/eventbus/EventBus.java	*add parameter*	EventSubscriber:wrapper
dispatch( )	/eventbus/EventBus.java	*remove parameter*	EventHandler:handler
dispatch( )	/eventbus/EventBus.java	*remove parameter*	EventHandler:wrapper

#### Case2: Example of consistent refactoring

**Fig 9:**
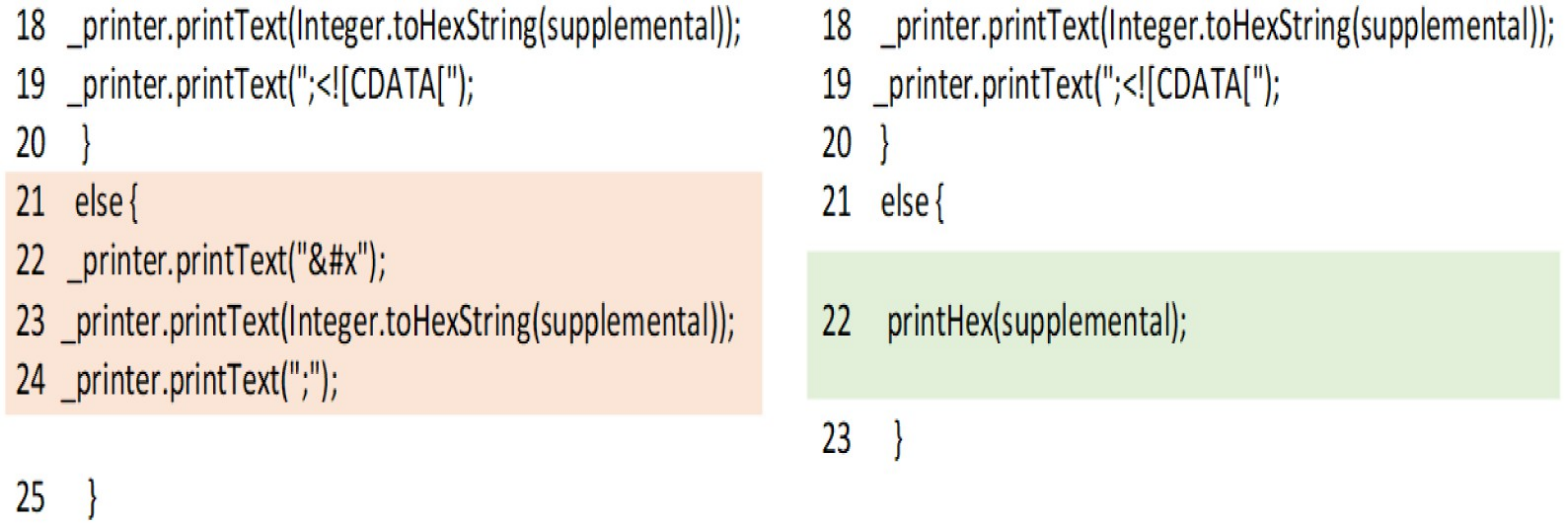
Example of consistent clone refactoring in Xerces_J.

In Xerces_J, Version 2.5.0, there is a clone class with two instances consisting of two variations of surrogates( ) method in files ‘/org/apache/xml/serialize/BaseMarkupSerializer.java’ and ‘/org/apache/xml/serialize/XML11Serializer.java’. Extract-method refactoring pattern is applied consistently on both methods and cloned code is extracted to a new method named printHex (supplemental) as shown in Fig 9. This new method is then called from the original methods. Our analysis of this refactoring example reveals that this clone class was not removed even after clone removal refactorings are applied consistently on it. Source code analysis of the clone class shows that length of cloned code fragments in both methods is 30 source lines of code whereas *extract method* refactoring is applied on only three lines of code. The source code of both the clone fragments are also very similar (except some checks which validates XML data) which indicates that more refactorings can also be performed on these cloned code fragments. This example represents that developers were aware of the existence of clones but they applied refactoring partially on cloned code. This may also represents that long clone code fragments may remain in the system even if clone removal refactorings are applied on all code fragments of the clone class. This example also shows that *extract method* refactoring can only be applied on identical clones. Clones that are slightly different may not be removed easily. Developers has to manage them in versions.

#### Case 3: Example of consistent and inconsistent refactoring

In Guava, there is a clone class with two instances waitInterruptibly ( ) and waitUnInterruptibly () in class Monitor.java. Three refactorings are performed on this clone class. Rename-method refactoring is applied on both instances of clone class and changed the method names from waitInterruptibly ( ) to await ( ) and waitUnInterruptibly () to awaitUnInterruptibly (). Third refactoring task is remove control flag which is applied only on one clone instance. In this example, although *add parameter* refactoring is consistently applied on both instances but it changed the method names differently which changed their textual similarity. Moreover one inconsistent refactoring also made them different from each other and they did not remain similar enough to be detected as clones of each other in next version.

### Understanding software’s characteristics through clone refactoring analysis

We analyze clone refactorings for each system to understand system characteristics in terms of clone refactoring.

Average clone refactoring of JabRef is less than 1% (Table 3). However, these refactorings are consistent refactorings as compared to other systems except JHotDraw and also removed 30% of the clones from the system. This indicates that although less refactorings are applied on clones, yet developers are aware of cloning in the system when performing clone refactoring tasks as compared to other systems.

Average clone refactoring ratio of Guava and Xerces_J is almost same (less than 3%) whereas average clone removals are greater in Guava than Xerces_J. This indicates that intention of developers of Guava is for removing clones through refactoring. Consistent clone refactorings are also same for both systems i.e. 15–17% clones are consistently refactored.

Cloning in JFreeChart is greater than other systems and clone classes of JFreeChart are also larger in size than other systems but less refactorings are performed on them during the releases. Number of clone removal is also less than other systems. This indicates that trivial refactorings are applied on clones which did not remove cloning from the system. This may indicate that either developers are not aware of cloning in the system or not interested in refactoring clones. It also may be possible that clones are not refactorable.

In JHotDraw, greater number of clone refactorings are performed in versions than other systems and a larger percentage of the clones are removed as a result of those refactoring as compared to other systems. In JHotDraw, the refactorings are consistent as compared to other systems. Although, original architecture of JHotDraw is changed after 5th release but the system remains consistent in all the versions in terms of clone refactoring, clone removal and refactoring consistency. This indicates stability of JHotDraw and awareness of developers about cloning in the system and they refactor the code clones appropriately. Based upon these observations, a developer can assess the evolvability and stability of the JHotDraw for future releases.

## Threats to validity in clone refactoring study

In this section we discuss threats to validity that we identified in studying clone refactorings.

**External validity**
  Our subject systems are open source Java systems, some of which have been used for clone research. We selected these systems to avoid biasness of our study towards any specific software domain or size. As these systems are open source systems, where different developers freely contribute to the development of a project, it reduces the chances of biasness in terms of a developer’s programming style (e.g. copy paste approach).
  We took on an average 13 releases of each system to study clone evolution. These are the latest releases of these systems which represents a large time period of these systems. This is a limitation of our study that we did not analyze the systems for their entire lifetime. Research findings for entire lifetime of a system may be different but more than ten versions represent a large time period of a system which reduce the threat of generalizability for the entire lifetime.
  Moreover, these releases vary 1) in release type such as major and minor releases and also in 2) release time duration which reduces the threat of limiting the analysis to specific kinds of circumstances in which a certain clone evolution activity is performed and hence enhance the generalizibilty of the results for different types of releases.
**Internal validity**
  Exact explanations for the observations of a software system depends upon the complete domain knowledge of the software. Lack of domain knowledge may miss important insights during results analysis. Different factors such as number of bug fixes or new requirements in a software release may cause an increase/decrease in clone evolution patterns for that particular release. To address this threat we discussed the observations with the experts (experienced developers and senior research fellows) and consulted to the available resources such as source code, change repositories data and project documentation.
**Construct validity**
  Clone refactoring results depend upon clone data and refactoring data detected within a software release which again depends upon the clone detection tool and refactoring detection tool. For clone detection we used SourcererCC because precision and recall of the tools are better than other techniques. For detection of clone refactoring, we used RefFinder tool. Our clone refactoring analysis depends upon the data detected by other’s tools (SourcererCC and RefFinder) and this is a limitation of our study, however, results of RefFinder is manually validated by our experts (as we discussed in the section of experimental setup) and false positives are removed.

## Conclusions

In this paper, we performed an empirical study on code clone refactorings in versions of five software systems. To conduct the study in a systematic way, we defined evolution patterns of clone refactoring in a formal notation. Based on these patterns we extracted clone evolution data to perform longitudinal analysis of clone refactorings in software. Analysis of the results on five software systems shows that evolution analysis of clone refactorings is helpful for software mainteneace.

Evolution study of clone refactorings gives quantitative facts that lead to qualitative inferences/implications about the clones which will help developers in managing clones in practice. Our results showed that a small proportion of code clones are refactored during the releases however, most of the refactorings are performed before major releases. Most of the refactoring techniques applied on our studied systems are the refactorings whose purpose is to improve program comprehension and code structures which indicates that developers perform clone refactorings for the purpose of improving the quality of cloned code instead of removing them from the system. Our results also show that a small percentage of clone refactorings cause the clone removal during releases. Clone classes that are smaller in size are removed more often than larger clone classes. Analysis of clone removal refactorings reveal that there are various reasons of clone removal. In some cases, the reason is the type of refactorings such as clone removal refactorings are applied on all the clone instances of a clone class. Another reason includes frequent and inconsistent refactorings applied on clones such as some of the clone instances are refactored frequently with trivial refactoring types. Analysis of (in)consistent refactorings shows that most of the clones are refactored inconsistently in the versions of studied software systems which indicates one of the two cases. First, in many cases developers are not aware of cloning in the system and that they perform refactorings unintentionally. Second, different clone instances of a clone class require different types of refactorings. Our analysis of some consistent and inconsistent refactorings shows that in most of the cases of inconsistent refactorings, the types of refactoring performed are trivial refactorings. This may indicate that developers are less concerned about cloning while performing trivial refactorings.

We believe that our study on the evolution of clone refactorings will be helpful for developers in handling clones in industrial software and consequently in devising tools to support proper management of clones. In future, we will include subject systems of other languages to verify the generalizability of our findings regarding clone refactorings in software releases.

## Supporting information

S1 Dataset (RAR)

S1 Text Link for the dataset, tool and User manual is: https://github.com/j-kanwal/CRefEvol. (TXT)
